# Decline in Antibody Concentration 6 Months After Two Doses of SARS-CoV-2 BNT162b2 Vaccine in Solid Organ Transplant Recipients and Healthy Controls

**DOI:** 10.3389/fimmu.2022.832501

**Published:** 2022-02-23

**Authors:** Sebastian Rask Hamm, Dina Leth Møller, Laura Pérez-Alós, Cecilie Bo Hansen, Mia Marie Pries-Heje, Line Dam Heftdal, Rasmus Bo Hasselbalch, Kamille Fogh, Johannes Roth Madsen, Jose Juan Almagro Armenteros, Andreas Dehlbæk Knudsen, Johan Runge Poulsen, Ruth Frikke-Schmidt, Linda Maria Hilsted, Erik Sørensen, Sisse Rye Ostrowski, Zitta Barrella Harboe, Michael Perch, Søren Schwartz Sørensen, Allan Rasmussen, Henning Bundgaard, Peter Garred, Kasper Iversen, Susanne Dam Nielsen

**Affiliations:** ^1^Viro-Immunology Research Unit, Department of Infectious Diseases, Rigshospitalet, Copenhagen University Hospital, Copenhagen, Denmark; ^2^Laboratory of Molecular Medicine, Department of Clinical Immunology, Rigshospitalet, Copenhagen University Hospital, Copenhagen, Denmark; ^3^Department of Cardiology, Rigshospitalet, Copenhagen University Hospital, Copenhagen, Denmark; ^4^Department of Hematology, Rigshospitalet, Copenhagen University Hospital, Copenhagen, Denmark; ^5^Department of Cardiology, Herlev and Gentofte Hospital, Copenhagen University Hospital, Copenhagen, Denmark; ^6^Novo Nordisk Foundation Center for Protein Research, Faculty of Health and Medical Sciences, University of Copenhagen, Copenhagen, Denmark; ^7^Department of Clinical Biochemistry, Rigshospitalet, Copenhagen University Hospital, Copenhagen, Denmark; ^8^Department of Clinical Medicine, Faculty of Health and Medical Sciences, University of Copenhagen, Copenhagen, Denmark; ^9^The Blood Bank, Department of Clinical Immunology, Rigshospitalet, Copenhagen University Hospital, Copenhagen, Denmark; ^10^Department of Pulmonary and Infectious Diseases, Hospital of North Zealand, Copenhagen University Hospital, Hillerød, Denmark; ^11^Department of Nephrology, Rigshospitalet, Copenhagen University Hospital, Copenhagen, Denmark; ^12^Department of Surgical Gastroenterology, Rigshospitalet, Copenhagen University Hospital, Copenhagen, Denmark; ^13^Department of Emergency Medicine, Herlev and Gentofte Hospital, Copenhagen University Hospital, Copenhagen, Denmark

**Keywords:** SARS-CoV-2, COVID-19, vaccine, BNT162b2, solid organ transplant recipient, immunogenicity, vaccination

## Abstract

**Background:**

Previous studies have indicated inferior responses to severe acute respiratory syndrome coronavirus-2 (SARS-CoV-2) vaccination in solid organ transplant (SOT) recipients. We examined the development of anti-receptor-binding domain (RBD) immunoglobulin G (IgG) after two doses of BNT162b2b in SOT recipients 6 months after vaccination and compared to that of immunocompetent controls.

**Methods:**

We measured anti-RBD IgG after two doses of BNT162b2 in 200 SOT recipients and 200 matched healthy controls up to 6 months after first vaccination. Anti-RBD IgG concentration and neutralizing capacity of antibodies were measured at first and second doses of BNT162b2 and 2 and 6 months after the first dose. T-cell responses were measured 6 months after the first dose.

**Results:**

In SOT recipients, geometric mean concentration (GMC) of anti-RBD IgG increased from first to second dose (1.14 AU/ml, 95% CI 1.08–1.24 to 11.97 AU/ml, 95% CI 7.73–18.77) and from second dose to 2 months (249.29 AU/ml, 95% CI 153.70–385.19). Six months after the first vaccine, anti-RBD IgG declined (55.85 AU/ml, 95% CI 36.95–83.33). At all time points, anti-RBD IgG was lower in SOT recipients than that in controls. Fewer SOT recipients than controls had a cellular response (13.1% vs. 59.4%, p < 0.001). Risk factors associated with humoral non-response included age [relative risk (RR) 1.23 per 10-year increase, 95% CI 1.11–1.35, p < 0.001], being within 1 year from transplantation (RR 1.55, 95% CI 1.30–1.85, p < 0.001), treatment with mycophenolate (RR 1.54, 95% CI 1.09–2.18, p = 0.015), treatment with corticosteroids (RR 1.45, 95% CI 1.10–1.90, p = 0.009), kidney transplantation (RR 1.70, 95% CI 1.25–2.30, p = 0.001), lung transplantation (RR 1.63, 95% CI 1.16–2.29, p = 0.005), and *de novo* non-skin cancer comorbidity (RR 1.52, 95% CI, 1.26–1.82, p < 0.001).

**Conclusion:**

Immune responses to BNT162b2 are inferior in SOT recipients compared to healthy controls, and studies aiming to determine the clinical impact of inferior vaccine responses are warranted.

## Introduction

Solid organ transplant (SOT) recipients receive immunosuppressive therapy to prevent allograft rejection, leaving them at higher risk of infections and infection-related morbidity and mortality (1). This may also be true for coronavirus disease 2019 (COVID-19) (2–6).

Vaccines are the mainstay of preventive efforts to curb the COVID-19 pandemic. The BNT162b2 vaccine evokes both humoral and cellular responses in immunocompetent populations (7, 8) and demonstrated excellent efficacy in a Phase 3 trial. However, SOT recipients were not included in these trials (7–9). Reports on the short-term response to severe acute respiratory syndrome coronavirus-2 (SARS-CoV-2) mRNA vaccines in SOT recipients indicate that the humoral response after two doses of vaccine (5, 6, 10–18) is inferior to what was initially reported in immunocompetent populations (7, 8).

The neutralizing capacity of SARS-CoV-2 antibodies describes the ability of antibodies to prevent the viral spike (S) protein receptor-binding domain (RBD) from interacting with the angiotensin-converting enzyme II (ACE-II) receptor, thus preventing viral cell entry and infection. A previous study found that the neutralizing capacity of antibodies predicted protection from symptomatic infection in an immunocompetent population (19). Data on neutralizing capacity and cellular responses after SARS-CoV-2 mRNA vaccination in SOT recipients are scarce and conflicting (13, 17, 20–23).

It has been shown that antibodies after both SARS-CoV-2 infection and vaccination decline over time in immunocompetent populations (24–26). However, to date, there is only one report on the kinetics of antibodies after vaccination in SOT recipients with 6 months of follow-up (27) without assessment of neutralizing capacity of antibodies, cellular immunity, and a control group. To provide further knowledge on immune responses to the BNT162b2 vaccine in SOT recipients, we aimed to investigate the antibody responses in the first 6 months after vaccination with two doses of BNT162b2 in a large cohort of SOT recipients and compare the antibody responses to that in healthy controls. Furthermore, we determined the neutralizing capacity of antibodies and cellular responses. Finally, we determined risk factors for humoral non-response.

## Methods

### Study Design

In this prospective observational cohort study, all adult liver, kidney, and lung transplant recipients followed at Copenhagen University Hospital, Rigshospitalet, who had not yet received their second dose of SARS-CoV-2 vaccine, were invited to participate. First round of inclusion took place from January through April 2021. From July 2021, all adult liver, kidney, and lung transplant recipients who were not already included were invited to participate regardless of vaccination status. Healthy controls were recruited from a parallel study of healthcare workers at two university hospitals in greater Copenhagen (Rigshospitalet and Herlev-Gentofte Hospital) (28). Controls were included from December 2020 through April 2021 (28). All participants received two doses of BNT162b2 as part of the Danish vaccination program. Participation in the study was voluntary and did not interfere with the vaccination strategy.

The presence of antibodies against SARS-CoV-2 nucleocapsid (N) protein was used as a proxy for previous infection, and N-antibody-positive participants were excluded from further analyses. To compare vaccine responses in SOT recipients and controls, N-antibody-negative SOT recipients were matched on age and sex (1:1) to N-antibody-negative controls.

The study was conducted in accordance with the Declaration of Helsinki. All SOT recipients and controls provided informed oral and written consent. The study was approved by the institutional review board at Regional Scientific Ethics Committee of the Capital Region of Denmark (H-20079890).

### Blood Sampling

Participants were followed with repeated blood sampling up to 6 months after the first vaccine dose; a *baseline sample* was collected either before or up to 13 days after the first dose of BNT162b2 vaccine. A *3-week sample* was collected from 14 days and up to 33 days after the first dose of BNT162b2 and before administration of the second dose, a *2-month sample* was collected from 34 days and up to 90 days after the first dose and only after administration of the second dose, a *6-month sample* was collected from 91 days after the first dose and up to 273 days after the first dose and only after administration of the second dose. Samples for assessment of cellular responses were collected 6 months after the first vaccination dose.

### Clinical Information

Clinical information on SOT recipients, including demographics, transplant type, positive SARS-CoV-2 PCR test, comorbidities (cardiovascular disease, chronic pulmonary disease, diabetes, and *de novo* non-skin cancer), rejections, and immunosuppressive medication, was collected from medical records. Data regarding vaccination status were collected from the Danish Vaccination Register (29). Furthermore, all SOT recipients and controls were asked to complete a questionnaire including questions about height and weight.

### Determination of Antibodies

Venous blood was collected in serum separator tubes and centrifuged at 1,800 G. Serum was stored at -80°C until use.

Titers of immunoglobulin G (IgG) antibodies specific for the RBD of the S protein were determined using an in-house ELISA-based assay as described previously (30). In brief, Nunc Maxisorp 384-well plates (Thermo Fisher Scientific, MA, USA) were coated with purified recombinant RBD overnight in phosphate-buffered saline (PBS; Rigshospitalet, Copenhagen, Denmark). Then, the wells were blocked for 1 h with PBS and Tween 20 (PBS-T, Merck, Darmstadt, Germany), and diluted patient serum was added. Hereafter, horseradish peroxidase (HRP)-conjugated polyclonal rabbit-anti-human IgG (Agilent Technologies, Santa Clara, CA, USA) was added. Tetramethylbenzidine (TMB) ONE substrate (Kem-En-Tec, Taastrup, Denmark) was added to react with the HRP, and the reaction was stopped by adding H_2_SO_4_. Optical density was measured at 450–630 nm using a Synergy HT absorbance reader (Biotek). Between each step, plates were washed four times with PBS-T. Recombinant human IgG antibody anti-S1 protein (HC2001, Genscript Biotech, New Jersey, USA) was used as a calibrator. Interpolated IgG titers were given in arbitrary units (AU)/ml. A value above 1 AU/ml was considered detectable, and samples with a value below limit of detection were given the value of 1 AU/ml.

To measure antibodies specific for the SARS-CoV-2 N antigen, the Elecsys^®^ Anti-SARS-CoV-2 immunoassay (Roche Diagnostics GmbH, Germany) and a Cobas 8000 analyzer system (Roche Diagnostics) were used according to the manufacturer’s instructions.

We used an in-house ELISA to estimate neutralizing capacity of antibodies, as previously described (31). Briefly, Nunc Maxisorp 96-well plates were coated with ACE-II ectodomains in PBS overnight. Hereafter, patient serum was incubated with a solution of biotinylated recombinant RBD and Pierce high-sensitivity streptavidin-HRP (Thermo Fisher Scientific) for 1 h in non-binding 96-well plates and then added to the ACE-II ectodomain-coated wells for 35 min. Plates were developed as described above. After each step, the wells were washed three times with PBS-T. This in-house ELISA-based estimation of inhibition of the ACE-II host receptor/RBD interaction correlates well with the gold standard plaque reduction neutralization test (R = 0.9231) (31).

### Interferon-Gamma Releasing Assay

To assess the cellular response 6 months after the first dose of vaccine, we measured interferon-γ (IFN-γ) release after stimulation of T cells in fresh whole blood with SARS-CoV-2 S1 antigen using a commercial kit according to manufacturer’s instruction (product EQ 6841-9601, EUROIMMUN). In brief, 4 ml of venous blood was collected in lithium-heparin-coated tubes and aliquoted to three different tubes: One blank tube to measure unstimulated IFN-γ concentration, one tube containing SARS-CoV-2 S1 protein-specific peptides, and one tube containing a mitogen serving as a positive control (product ET 2606-3003, EUROIMMUN, Lübeck, Germany). After incubation for 21 h at 37°C, samples were centrifuged at 12,000 G for 10 min. IFN-γ concentrations were measured using an IFN-γ ELISA kit. Results from the unstimulated tubes were subtracted from the SARS-CoV-2 S1 peptide and mitogen tubes to estimate IFN-γ concentrations caused by SARS-CoV-2 S1 stimulation of T cells.

### Definitions

A positive humoral response was defined as having both a minimum of 25% inhibition in the neutralizing assay and concurrent anti-RBD IgG >225 AU/ml (28, 32).

A positive T-cell response was defined as an IFN-γ concentration above 200 mIU/ml in a stimulated sample after subtracting IFN-γ concentration in the corresponding negative control, as per manufacturer’s instructions.

### Statistics

Continuous data were reported with means and standard deviations (SDs) or medians with interquartile range (IQR), as appropriate. Differences in continuous variables were assessed by Student’s t-test or Mann–Whitney U test, as appropriate. Categorical data were reported as frequency counts and percentage of subjects within each category. Independence was tested using the χ^2^ test or Fisher’s exact test, as appropriate. Furthermore, characteristics of SOT patients grouped by transplanted organ were compared using ANOVA or Kruskal–Wallis test when appropriate. Normality of data was assessed by quantile-quantile plots.

To compare changes in antibody concentrations and neutralizing capacity between SOT recipients and controls, we fitted a two-part mixed linear model with either log-transformed anti-RBD IgG concentrations or neutralizing capacity as dependent value and sample time and SOT recipient/control status as fixed effects. To compare changes in antibody concentration and neutralizing capacity between type of transplanted organ within the SOT recipients, we fitted a two-part linear mixed-effects model with either log-transformed anti-RBD IgG concentrations or neutralizing capacity as dependent variable and sample time and organ type as fixed effects. The two-part mixed model is composed of a zero-inflation model, which models the probability of an observation being zero, and a conditional model, which models the anti-RBD IgG concentration or neutralizing level for non-zero observations (33). The zero-inflation model was necessary due to the bimodality present in the data; there was a high proportion of observations with no detectable levels of anti-RBD IgG or neutralizing antibodies. To visualize predicted and observed antibody concentration and neutralizing capacity, geometric mean concentration (GMC) of anti-RBD IgG or mean neutralizing capacity with 95% CI at each visit, as predicted by the model, was plotted on top of observed anti-RBD IgG concentration or neutralizing capacity for each sample. Furthermore, changes in log-transformed antibody concentration with time since first vaccination as a continuous variable was modeled using cubic splines. To accommodate the bimodality of the data, a two-part model with a zero-inflation model and a conditional model was used, as was the case with the mixed linear model. The model fitted log-transformed antibody concentration dependent on time since first vaccination and SOT recipient/control status and allowed for interaction between time since vaccination and SOT recipient/control status. Knots were defined as days 19, 52, and 154, which were the 25%, 50%, and 75% quantile of days from vaccination. The model was plotted on top of the observed anti-RBD IgG concentration for each sample. Samples collected before the day of first vaccination was coded as collected at day of vaccination. The decline in anti-RBD IgG GMC in SOT recipients and controls from the day of maximum concentration to day 180 after vaccination was calculated by exponentiating the difference in log-transformed anti-RBD IgG concentration predicted by the natural cubic spline model.

Univariable and multivariable Poisson regression with robust standard error estimator was performed to test for associations between either humoral or cellular response to BNT162b2 vaccine and independent variables in SOT recipients after the 6-month sample. In the multivariable model, we adjusted for sex and age.

To test for the correlation between IFN-γ concentration and anti-RBD IgG concentration in SOT recipients with results from the interferon gamma releasing assay, Spearman’s test was performed.

The number of samples from each group at each time point is represented in **Table S1** along with a table of missing data on body mass index (BMI). We chose to perform mixed linear models to accommodate for the potential of missing samples at some time points.

A p-value <0.05 was considered significant. Statistical analyses were performed using Rstudio Version 1.2.5001 (R Core Team, 2020, Vienna, Austria).

## Results

### Clinical Characteristics of Solid Organ Transplant Recipients

We included 200 SOT recipients and 200 age- and sex-matched controls (**Table 1**). The median age of the SOT recipients was 57 years (IQR 50-64) and 110 (55.0%) were male. Age, sex, and BMI were similar for SOT recipients and controls, but median time between first and second vaccine dose was shorter in SOT recipients than that in controls (22 days vs. 30 days, p < 0.001) (**Table 1**).

**Table 1:** Characteristics of SOT recipients and controls.

	SOT recipients	Controls	p-value
N	200	200	
Median age, years (IQR)	57.0 (49.8–64.0)	56.5 (49.0–62.0)	0.172
Male gender, n (%)	110 (55%)	114 (57%)	0.763
Median time between first and second vaccine dose days (IQR)	22 (21–23)	30 (29–32)	< 0.001
BMI, mean (SD)	26.1 (5.5)	25.8 (4.5)	0.656
Median time from first vaccination to sample, days (IQR)			
-Baseline	-19.0 (32.8-1.0)	0.0 (0.0–0.0)	< 0.001
-Three weeks	21.0 (19.0–22.0)	23.0 (21.0–27.0)	< 0.001
-two months	59.0 (52.0–64.0)	61.0 (57.0–65.0)	0.015
-Six months	168 (154.0–200.0)	165.0 (156.0–200.9)	0.693

IQR, Interquartile range; SD, standard deviation.

The SOT recipients consisted of 61 (30.5%) liver, 102 (51.0%) kidney, and 37 (18.5%) lung transplant recipients (**Table 2**). Among SOT recipients, 153 (76.5%) had comorbidities; the distribution of comorbidities is shown in **Table 2**. The median time from transplantation to vaccination was 5.8 years (IQR 2.4–10.5), and 27 (13.5%) SOT recipients were less than 1 year from transplantation. Maintenance immunosuppression is shown in **Table 2**. Three patients (1.5%) received high-dose methylprednisolone treatment for acute rejection less than 90 days prior to vaccination.

**Table 2:** Characteristics of SOT recipients.

	All recipients (n = 200)	Liver transplant recipients n = 61 (30.5%)	Kidney transplant recipients* n = 102 (51.0%)	Lung transplant recipients** n = 37 (18.5%)	p-value
Age, median (IQR)	57.0 (49.8–64.0)	55 (48.00–63.00)	57.0 (51.00–64.00)	59 (53.00–66.00)	0.103
Sex (male), n (%)	110 (55.0%)	36 (59.0%)	58 (56.9%)	16 (43.2%)	0.272
BMI (kg/m^2^), mean (SD)	26.05 (5.46)	27.11 (5.78)	25.68 (4.82)	23.59 (5.22)	0.072
Comorbidities, % (n)					
-Cardiovascular disease	139 (69.5%)	20 (32.8%)	90 (88.2%)	29 (78.4%)	< 0.001
-Chronic pulmonary disease	20 (10.0%)	8 (13.1%)	7 (6.8%)	5 (13.5%)	0.306
-Diabetes mellitus	45 (22.5%)	15 (24.6%)	23 (22.5%)	7 (18.9%)	0.809
-*De novo* non-skin cancer ***	6 (3.0%)	0 (0.0%)	5 (4.9%)	1 (2.7%)	0.268
Time since transplantation, median (IQR), years	5.8 (2.4–10.5)	5.4 (2.7–10.2)	6.4 (2.3–10.6)	5.6 (1.8–10.2)	0.871
Transplanted < 1 year before vaccination n (%)	27 (13.5%)	6 (9.8%)	16 (15.7%)	5 (13.5%)	0.623
Immunosuppressive treatment					
-Azathioprine	31 (15.5%)	7 (11.5%)	19 (18.6%)	5 (13.5%)	0.443
-Mycophenolate	141 (70.5%)	45 (73.8%)	73 (71.6%)	23 (62.2%)	0.448
-Calcineurin inhibitor (Ciclosporin, Tacrolimus)	181 (90.5%)	54 (88.5%)	90 (88.2%)	37 (100.0%)	0.061
-mTOR inhibitor (Sirolimus, Everolimus)	29 (14.5%)	6 (9.8%)	12 (11.8%)	11 (29.7%)	0.023
-Corticosteroids	146 (73.0%)	28 (45.9%)	88 (86.3%)	30 (81.1%)	< 0.001
High dose methyl-prednisone rejection treatment < 90 days before vaccination, % (n)	3 (1.5%)	0 (0.0%)	1 (1.0%)	2 (5.4%)	0.168

*Including one kidney-pancreas transplant recipient. **Including two heart-lung transplant recipients, *** All SOT recipients with de novo non-skin cancer were treated with calcineurin inhibitors IQR, interquartile range; SD, standard deviation; mTOR, mammalian target of rapamycin.

### Geometric Mean Concentration of Anti-Receptor-Binding Domain Immunoglobulin G

**Figure 1:**
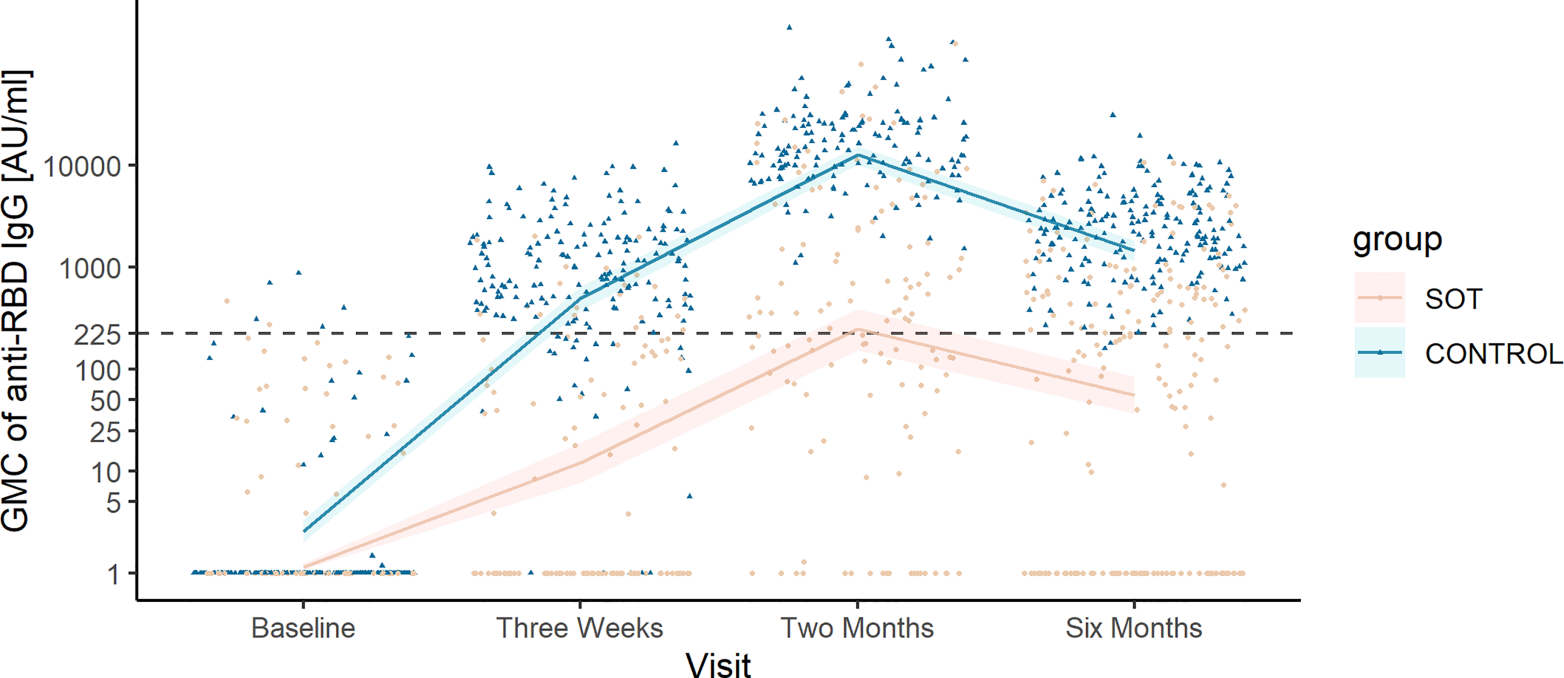
Observed anti-receptor-binding domain (RBD) immunoglobulin G (IgG) concentrations and predicted geometric mean concentration (GMC) of anti-RBD in solid organ transplant (SOT) recipients and controls. Development in predicted GMC of anti-RBD IgG represented in AU/ml plotted on top of the observed individual concentration of anti-RBD IgG at each sample time from SOT recipients (yellow) and controls (blue). The dashed horizontal line indicates the minimum threshold for positive humoral response.

In SOT recipients, the predicted GMC of anti-RBD IgG increased from baseline (1.14 AU/ml, 95% CI 1.08–1.24) to 3 weeks after the first vaccine dose (11.97 AU/ml, 95% CI 7.73–18.77) and from 3 weeks to 2 months after the first vaccine dose after receiving the second dose (249.29 AU/ml, 95% CI 153.70–385.19; **Figure 1**). From 2 to 6 months after the first vaccine, there was a significant decline in the predicted GMC of anti-RBD IgG to 55.85 AU/ml (95% CI 36.95–83.33).

**Figure 2:**
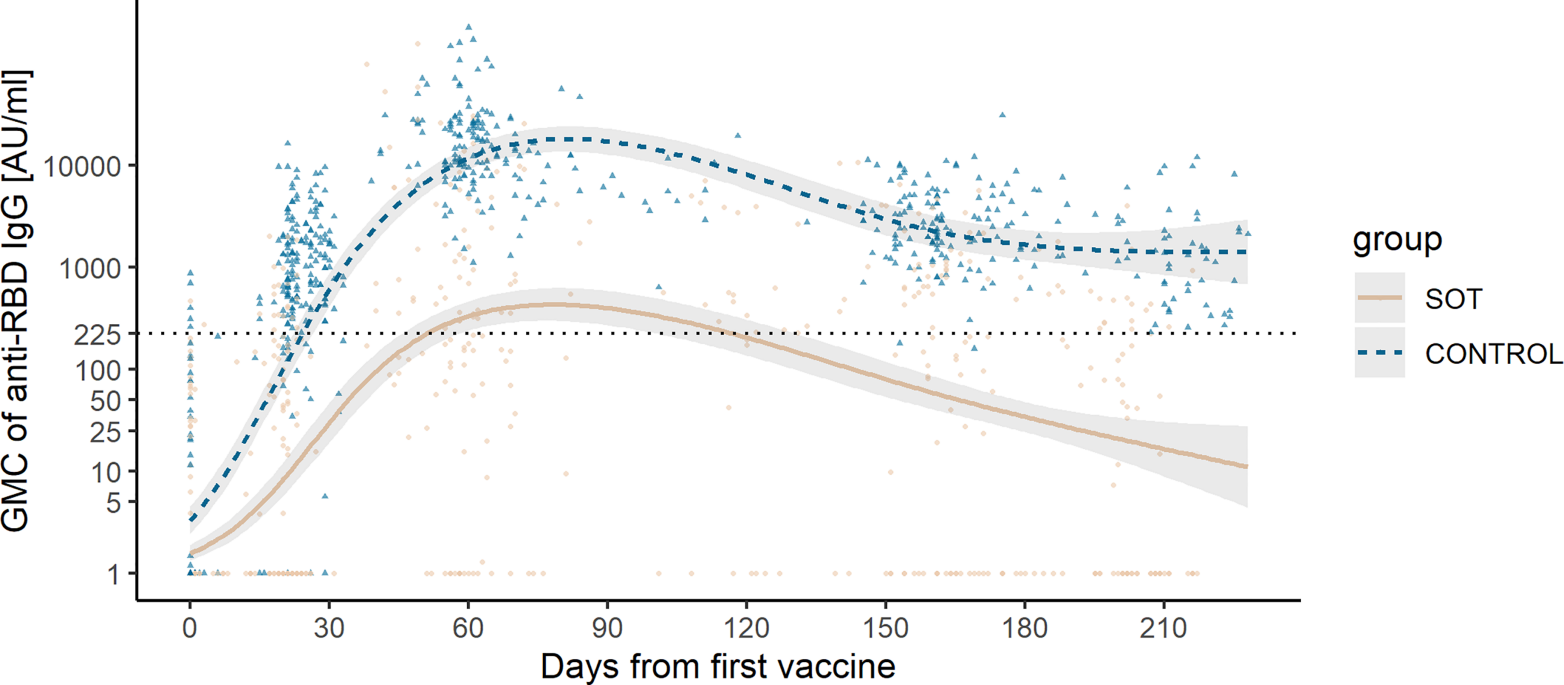
Observed anti-receptor-binding domain (RBD) immunoglobulin G (IgG) concentration and predicted geometric mean concentration (GMC) of anti-RBD IgG in solid organ transplant (SOT) recipients and controls. Development in predicted anti-RBD IgG concentration represented in AU/ml plotted on top of the observed individual concentration of anti-RBD IgG from the day of the first vaccination to 228 days after the first vaccination from SOT recipients (yellow) and controls (blue). The dashed horizontal line indicates the minimum threshold for positive humoral response.

The predicted GMC of anti-RBD IgG in SOT recipients was lower than that in controls at all time points (**Figure 1**). The kinetics of anti-RBD IgG GMC decline differed slightly between SOT recipients and controls. SOT recipients reached maximum antibody concentration 78 days after first vaccination, while this was the case for controls 81 days after first vaccination. The anti-RBD IgG GMC declined by 92.0% (95% CI 91.9–92.3) from maximum at day 78 to day 180 after first vaccination in SOT recipients. In controls, anti-RBD IgG GMC declined by 90.8% (95% CI 90.4–91.2) from maximum at day 81 to day 180 after first vaccination (**Figure 2**).

### Neutralizing Antibodies

**Figure 3:**
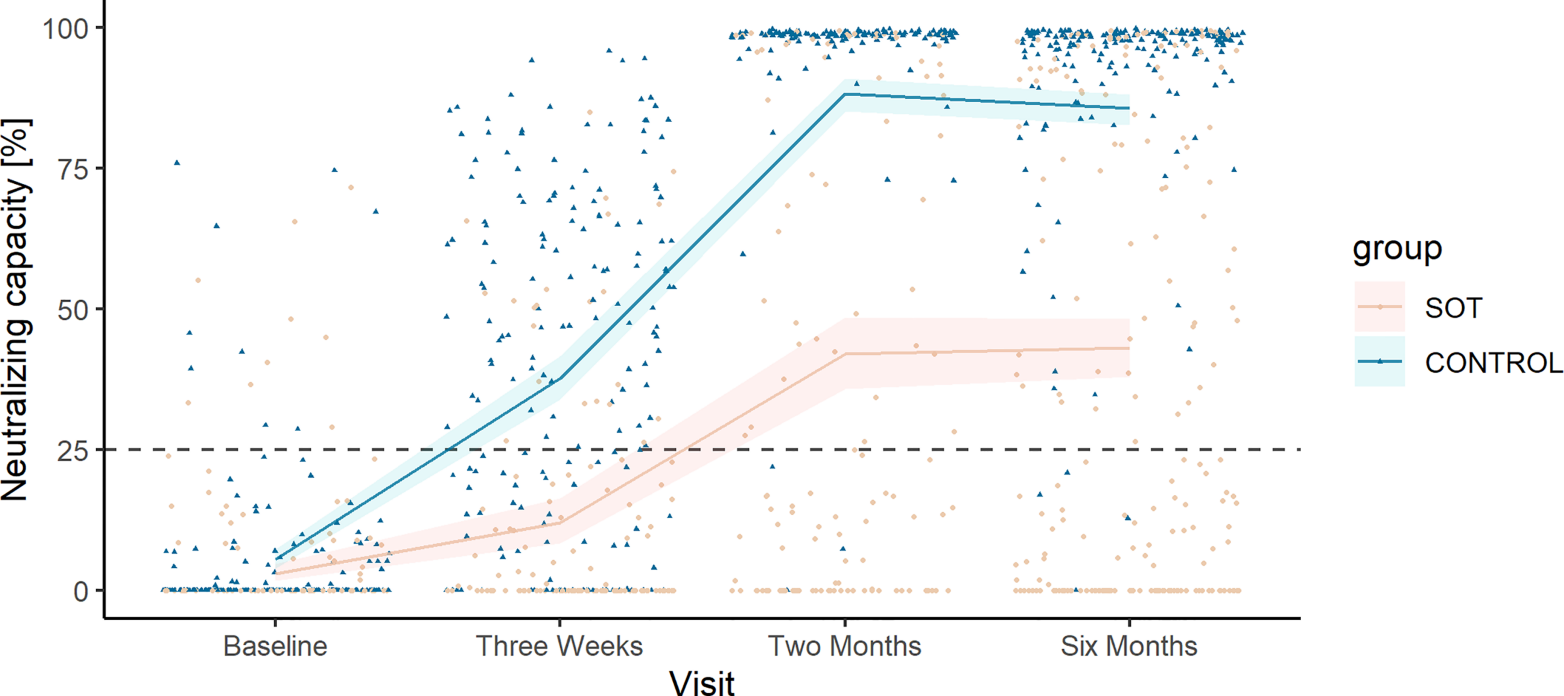
Observed neutralizing capacity and predicted mean neutralizing capacity in solid organ transplant (SOT) recipients and controls. Development in predicted mean neutralizing capacity represented in percent plotted on top of the observed individual neutralizing capacity at each sample time from SOT recipients (yellow) and controls (blue). The dashed horizontal line indicates the minimum threshold for positive humoral response.

In SOT recipients, the predicted mean neutralizing capacity increased from baseline (2.99%, 95% CI 1.77%–4.72%) to 3 weeks after the first vaccine dose (11.95%, 95% CI 8.30%–16.27%) and from 3 weeks after the first dose to 2 months after the first dose, 41.99% (95% CI 35.71%–48.19%, **Figure 3**). Six months after the first vaccine dose, the predicted mean neutralizing capacity in SOT recipients was 43.09% (95% CI 37.93%–48.27%).

The mean neutralizing capacity in SOT recipients was lower than that in controls at all time points after the first vaccine dose (**Figure 3**).

### Risk Factors Associated With Humoral Non-Response 6 Months After the First Vaccine Dose

Six months after the first vaccine dose, 72 of 200 (36%) SOT recipients had a positive humoral response, while this was the case for 195 of 200 controls (97.5%), p < 0.001.

**Figure 4:**
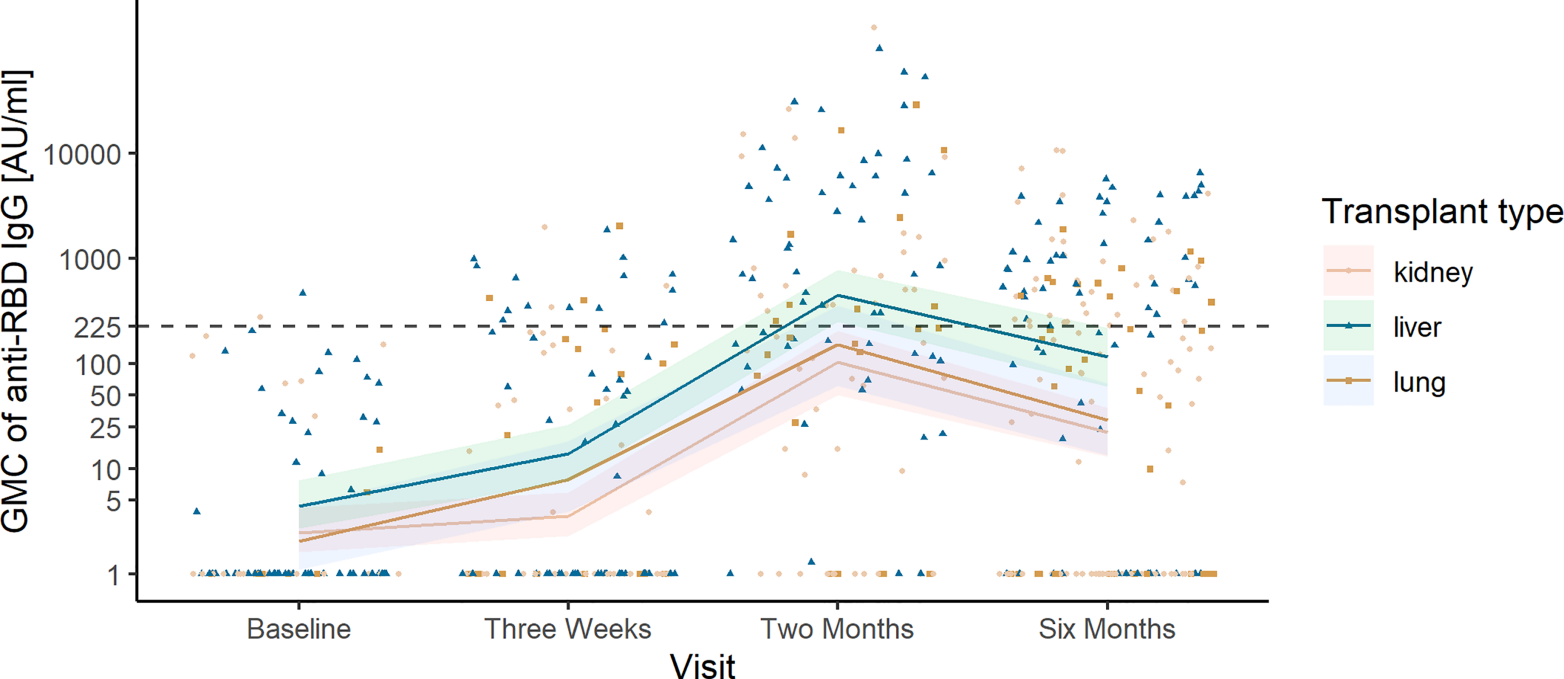
Observed anti-receptor-binding domain (RBD) immunoglobulin G (IgG) concentrations and predicted geometric mean concentration (GMC) of anti-RBD in liver, kidney, and lung transplant recipients. Development in predicted GMC of anti-RBD IgG represented in AU/ml plotted on top of the observed individual concentration of anti-RBD IgG at each sample time in liver (green/triangle), kidney (yellow/circle), and lung transplant recipients (brown/square). The dashed horizontal line indicates the minimum threshold for positive humoral response.

**Figure 5:**
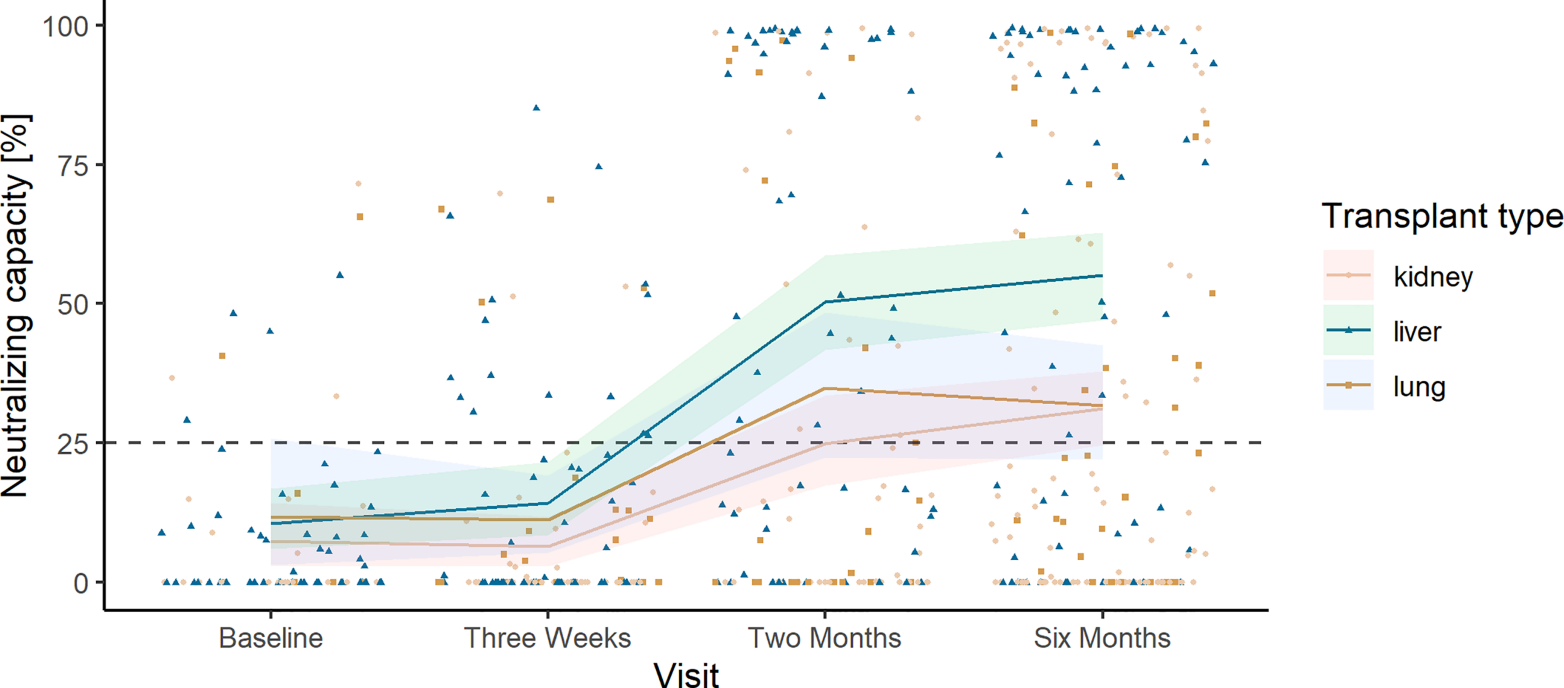
Observed neutralizing capacity and predicted mean neutralizing capacity in liver, kidney, and lung transplant recipients. Development in predicted mean neutralizing capacity represented in percent plotted on top of the observed individual neutralizing capacity at each sample time from liver (green/triangle), kidney (yellow/circle), and lung transplant recipients (brown/square). The dashed horizontal line indicates the minimum threshold for positive humoral response.

In SOT recipients, we found increasing age, being less than 1 year from transplantation, use of mycophenolate or corticosteroids, kidney or lung transplantation, and *de novo* non-skin cancer to be associated with an increased relative risk (RR) of humoral non-response after adjusting for sex and age (**Table 3**). Increased age was associated with increased RR of humoral non-response in all multivariable models. Furthermore, we found liver transplant recipients to have higher predicted anti-RBD GMC and neutralizing capacity than lung and kidney transplant recipients 6 months after the first vaccination (**Figures 4**, **5**).

**Table 3:** Risk factors of humoral non-response 6 months after the first vaccine dose.

	Univariable Risk Rate (95% CI)	p-value	Multivariable Risk Rate (95% CI) Sex + age + X	p-value
Age per 10 years	1.23 (1.11–1.35)	< 0.001		
Sex (male)	0.90 (0.73–1.10)	0.311		
BMI per increase in BMI (kg/m^2^)	1.00 (0.96–1.04)	0.968	1.00 (0.96–1.04)	0.981
Transplanted < 1 year before vaccination	1.56 (1.32–1.83)	< 0.001	1.55 (1.30–1.85)	< 0.001
Immunosuppressive treatment				
• No antimetabolite	Reference	Reference	Reference	Reference
• Azathioprine	0.84 (0.50–1.42)	0.519	1.01 (0.62–1.64)	0.967
• Mycophenolate	1.31 (0.91–1.88)	0.142	1.54 (1.09–2.18)	0.015
• No corticosteroids	Reference	Reference	Reference	Reference
• Corticosteroids	1.38 (1.04–1.85)	0.027	1.45 (1.10–1.90)	0.009
• Calcineurin inhibitor (Ciclosporin, Tacrolimus)	Reference	Reference	Reference	Reference
• mTOR inhibitor (Sirolimus, Everolimus)	0.85 (0.60–1.20)	0.350	0.87 (0.62–1.22)	0.435
Type of organ transplanted				
• Liver	Reference		Reference	
• Kidney	1.79 (1.3–2.48)	<0.001	1.70 (1.25–2.30)	0.001
• Lung (and heart-lung)	1.85 (1.3–2.63)	0.001	1.63 (1.16–2.29)	0.005
Comorbidities				
• Cardiovascular disease	1.37 (1.05–1.80)	0.021	1.24 (0.97–1.58)	0.080
• Chronic pulmonary disease	1.02 (0.72–1.43)	0.921	0.92 (0.64–1.32)	0.662
• Diabetes mellitus	1.30 (1.06–1.59)	0.012	1.18 (0.97–1.44)	0.095
• *De novo* non-skin cancer	1.59 (1.43–1.77)	< 0.001	1.52 (1.26–1.82)	< 0.001

BMI, body mass index; mTOR, mammalian target of rapamycin.

### T-Cell Response

The interferon gamma releasing assay was performed in a subset of 99 random SOT recipients 6 months after the first vaccine dose. In the control group, results from the interferon gamma releasing assay was available from 37 participants 6 months after the first vaccine dose. A total of 13 (13.1%) of the SOT recipients and 22 (59.4%) controls had a positive T-cell response (p < 0.001). Seven of 70 (10.0%) SOT recipients who did not develop a humoral response developed a T-cell response, while six of 29 (20.7%) SOT recipients who developed a humoral response developed a T-cell response. Log-transformed anti-RBD IgG concentration correlated with log-transformed IFN-γ concentration in SOT recipients (Spearman r: 0.4, p < 0.001).

In SOT recipients, lower anti-RBD IgG concentration was associated with increased risk of T-cell non-response (RR = 1.40 per 1,000 AU/ml decrease, 95% CI 1.14–1.72, p = 0.002). However, none of the risk factors associated with increased risk of humoral non-response was associated with increased risk of T-cell non-response.

### Clinical Outcomes

Three months after collection of the last sample, data on PCR-confirmed SARS-CoV-2 infections were available from 156 of 200 SOT recipients. Five of the 156 SOT recipients had a PCR-confirmed SARS-CoV-2 infection within 3 months of collection of the last sample. The GMC of anti-RBD IgG was 5.1 AU/ml 6 months after the first vaccine dose in the five SOT recipients.

## Discussion

In this large prospective cohort study of humoral and cellular responses to BNT162b2 vaccine in SOT recipients and healthy controls with 6 months of follow-up, we found that both SOT recipients and controls demonstrated increases in anti-RBD IgG concentrations after the first and second doses. However, SOT recipients had inferior humoral and cellular responses to two doses of BNT162b6 vaccine at all time points investigated. From 2 to 6 months after the first vaccine, anti-RBD IgG concentration declined in both groups but remained higher in controls than in SOT recipients. Furthermore, we found increasing age, being less than 1 year from transplantation, treatment with mycophenolate or corticosteroids, being kidney or lung transplant recipient, and *de novo* non-skin cancer to be associated with humoral non-response.

Most previous studies have investigated short-term humoral responses up to 3 months after the first dose of SARS-CoV-2 mRNA vaccine in SOT recipients (5, 6, 10–18, 34) and reported response rates similar to what we found 2 months after the first vaccination. The present study expands current knowledge by presenting data on both anti-RBD IgG concentration, neutralizing capacity of antibodies, and T-cell responses in SOT recipients and population controls with long-term follow-up.

There are two previous studies with more than 3 months of follow-up. Boyarsky et al. (35) reported data with 4 months of follow-up after the first dose of BNT162b2 or mRNA-1273 vaccine. The study found 67% of SOT recipients to have anti-spike IgG antibodies in 4 months after the first dose (35). Alejo et al. (27) reported data with 7 months of follow-up after the first dose of BNT162b2 or mRNA-1273 vaccine and found 72% of SOT recipients to have anti-spike IgG antibodies. We found 36% of SOT recipients to have a positive humoral response 6 months after the first vaccine dose. The difference could be due to our threshold relying on both neutralizing capacity and concentration of anti-RBD IgG. Furthermore, we excluded participants with evidence of previous infection in the form of N-antibodies, while this was not the case in the studies by Boyarsky et al. and Alejo et al.

Importantly, while inferior immune responses in SOT recipients have been reported, little is known about risk factors for humoral and cellular non-response (5, 6, 10–18, 27, 34, 35). We found increasing age, being less than 1 year from transplantation, use of mycophenolate or corticosteroids, being kidney or lung transplant recipient, and *de novo* non-skin cancer to be associated with humoral non-response 6 months after the first vaccination. Increasing age, use of mycophenolate, and shorter time from transplantation to vaccination have previously been found to be associated with humoral non-response up to 3 months after the first vaccine dose (5, 6, 10, 11, 15, 16, 21). Likewise, liver transplant recipients have been found to have a lower risk of humoral non-response compared to other organ groups (11, 21). However, kidney and lung transplant recipients may have a larger burden of comorbidities, as the proportion with cardiovascular disease tended to be higher than that in liver transplant recipients. This may contribute to the findings. The use of corticosteroids or *de novo* non-skin cancer has not previously been found to be associated with humoral non-response.

The BNT162b2 vaccine evokes both humoral and cellular responses (7, 8). We found a cellular response in 13.1% of SOT recipients 6 months after the first vaccine dose, while 59.4% of controls had a cellular response. Previously, cellular response to SARS-CoV-2 mRNA vaccines in SOT recipients has been investigated in up to 3 months after the first vaccine dose. Cellular response rates between 16% and 86% have been reported (11, 13, 17, 21, 23). The lower cellular response rate we observed might be due to our sampling time being 6 months after the first vaccine dose, while previous studies performed assays on samples collected only up to 3 months after the first vaccine dose.

As no correlate of protection based on neither anti-RBD IgG concentration nor neutralizing capacity of antibodies exists, we defined a threshold of positive humoral response as 25% neutralizing capacity and IgG >225 AU/ml. This was based on a receiver operating characteristic curve analysis to estimate the optimal cutoff between naturally infected convalescent sera and sera from individuals obtained before 2020 (30). Neutralizing capacity of antibodies has been shown to be an important factor in protection against symptomatic infection (19) and severe disease (36), and continued investigation into possible correlations between antibody concentration, neutralizing capacity of antibodies, and breakthrough infections is warranted. The need for establishing a clinical correlate of protection is underlined by results from studies that found increasing titers and response rates in SOT recipients after a third vaccine dose and that some SOT recipients remain without a humoral response even after a third dose (37–41). As the increase in titers after a third dose could be utilized to counter waning titers in those with a poor/waning response, a clinical correlate of protection could optimize timing and prioritization of scarce vaccine supplies.

Our study had limitations. First, we did not have data from all time points from all participants; second, we only provide T-cell responses from a subset of SOT recipients and controls and only after 6 months. Furthermore, the study was not powered to investigate clinical protection from infection. The strengths of this study include long-term follow-up with data up to 6 months after vaccination in a well-described cohort of SOT recipients and matched population controls that all received two doses of the BNT162b2 vaccine. We applied a neutralizing assay and a T-cell assay on a subgroup of participants. Lastly, the N-antibody assay enabled us to differentiate between antibodies induced by infection and vaccination.

In conclusion, we found humoral and cellular responses to BNT162b2 6 months after vaccination to be inferior in SOT recipients compared to healthy controls. Importantly, antibody levels increased from first vaccine dose to 2 months but declined from 2 months to 6 months after the first vaccine dose. Increasing age, being less than 1 year from transplantation, use of mycophenolate or corticosteroids, being kidney or lung transplant recipient, and *de novo* non-skin cancer were found to be associated with humoral non-response. The clinical implications of both humoral and cellular non-response remain unclear, and efforts to establish a clinical correlate of protection is highly warranted in order to identify both patients at risk of infections and the optimal timing of potential booster doses, as antibodies in SOT recipients decline over time.

## Data Availability Statement

The data are not publicly available due to privacy or ethical restrictions. The data that support the findings of this study are available on reasonable request from the corresponding authors. Requests to access the datasets should be directed to susanne.dam.poulsen@regionh.dk.

## Ethics Statement

The studies involving human participants were reviewed and approved by the Regional Scientific Ethics Committee of the Capital Region of Denmark. The patients/participants provided their written and oral informed consent to participate in this study.

## Author Contributions

KI, HB, SN, and PG conceived and designed the study. LP-A, CH, JM, RF-S, and LMH performed the experiments. SH and JAA analyzed the data. SH, LDH, MP-H, DM, KF, RH, AK, JP, ZH, MP, SS, AR, HB, SN, and KI collected samples and clinical data. ES and SO were in charge of biobanking. SH drafted the article. All authors contributed to the article and approved the submitted version.

## Funding

This work was financially supported by grants from the Carlsberg Foundation (grant number CF20-0045) and the Novo Nordisk Foundation (grant number NFF205A0063505 and NNF20SA0064201).

## Conflict of Interest

The authors declare that the research was conducted in the absence of any commercial or financial relationships that could be construed as a potential conflict of interest.

## Publisher’s Note

All claims expressed in this article are solely those of the authors and do not necessarily represent those of their affiliated organizations, or those of the publisher, the editors and the reviewers. Any product that may be evaluated in this article, or claim that may be made by its manufacturer, is not guaranteed or endorsed by the publisher.

## Supplementary Material

Supplementary Table 1 Available samples from SOT recipients and controls and missing data on BMI and SARS-CoV-2 PCR tests.
