# Supplementary material for: Decline in Antibody Concentration 6 Months After Two Doses of SARS-CoV-2 BNT162b2 Vaccine in Solid Organ Transplant Recipients and Healthy Controls
Source: Front Immunol. 2022 Feb 23;13:832501. doi: 10.3389/fimmu.2022.832501 (PMC8905653; doi:10.3389/fimmu.2022.832501)
Supplement: Supplementary Table 1 — Available samples from SOT recipients and controls and missing data on BMI and SARS-CoV-2 PCR tests. [file Table_1.docx]

|  | SOT recipients | Controls |
| --- | --- | --- |
| N | 200 | 200 |
| Available samples |  |  |
| - Baseline | 82 | 195 |
| - Three-weeks | 119 | 182 |
| - Two-months | 123 | 145 |
| - Six-months | 200 | 200 |
| **Missing data on BMI** | 100 | 26 |
| **Missing data on SARS-CoV-2 PCR tests** | 44 | 200 |

Table S1 – Available samples from SOT recipients and controls and missing data on BMI and SARS-CoV-2 PCR tests

BMI, body mass index; SARS-CoV-2, Severe acute respiratory syndrome coronavirus 2; PCR, polymerase chian reaction
